# Proposed Geometrical Tool for Cases of Laterally Adapted Tibial Tubercle during Total Knee Replacement

**DOI:** 10.1155/2021/5244034

**Published:** 2021-08-07

**Authors:** Umaima R. Khairy, Sadiq J. Hamandi, Ahmed S. Abid Ali

**Affiliations:** ^1^Biomedical Engineering Department, College of Engineering, Al-Nahrain University, Baghdad, Iraq; ^2^Orthopedics Department, College of Medicine, Al-Nahrain University, Baghdad, Iraq

## Abstract

The alignment of tibial component in total knee replacement operation must be achieved in three planes to ensure optimum results. In coronal plane, the alignment depends on three anatomical landmarks. These landmarks are tibial tuberosity, leg shin, and midtalar point. In eastern community, people get used to sit cross-legged which causes additional tension in the quadriceps muscle which is attached distally to the tibial tuberosity. This tension causes adaptation of the tuberosity laterally. Tuberosity adaptation causes the three anatomical landmarks being not collinear. In this work, eight cases of lateral adapted tubercle were diagnosed of this condition before the surgery and their X-ray images after the surgery were checked regarding tibial alignment. Tibial alignment has been checked by measuring the medial proximal tibial angle (MPTA) which is the angle between the mechanical tibial axis and the tibial component plateau. MPTAs for the eight cases were (86.9°–93.6°). Three cases had MPTA less than 90° indicating varus alignment and five of them had MPTA more than 90° indicating valgus alignment. A geometrical tool was designed using the DesignSpark Mechanical software as a proposed solution to solve the adaptation problem. The tool can give a method for fixing the tibial component precisely without any varus\valgus malalignment.

## 1. Introduction

**Figure 1:**
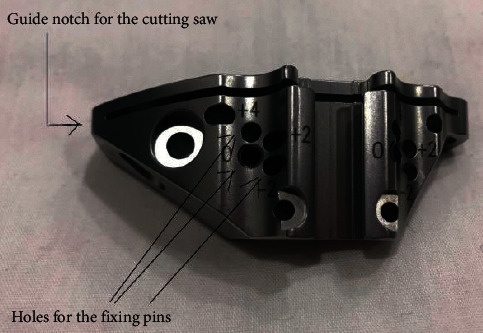
Tibial cutting blocks for left knees.

During total knee replacement surgery, in order to resect the tibial plateau correctly, a block should be placed satisfactorily in three planes near the tibial plateau. Then, the block would act as a saw guide to make the cut. The block for the left joint is shown in Figure 1 with the surgical saw notch also illustrated.

Ensuring correct placement of the block necessitates using some anatomical landmarks and anatomical aspects regarding the three planes (sagittal, coronal, and transverse planes), as in Table 1.

**Figure 2:**
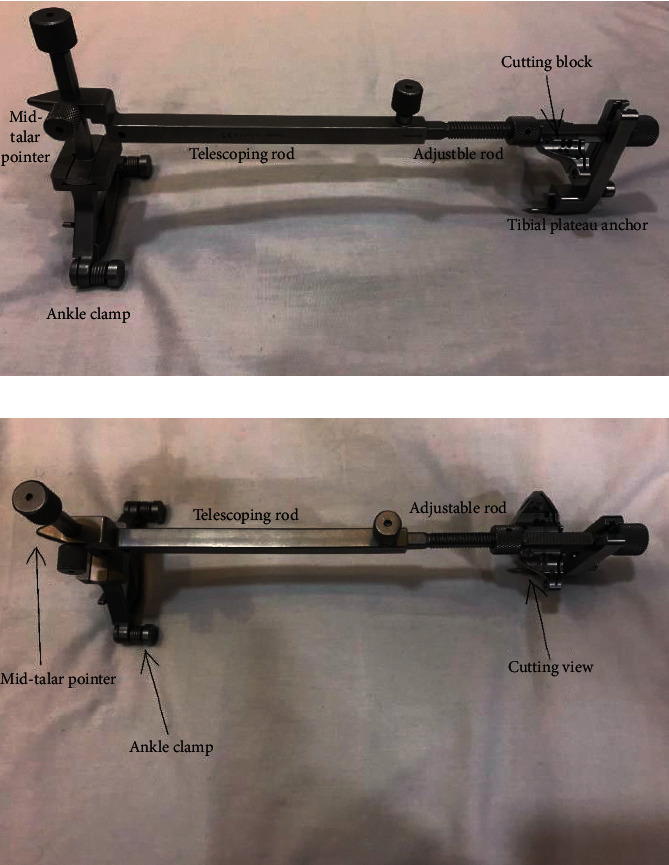
The extramedullary cutting guide. (a) Side view. (b) Top view.

The block is held by the extramedullary (EM) guide; then, placing the extramedullary guide correctly would ensure correct block placement. The extramedullary guide is shown in Figure 2.

Laterally adapted tibial tubercle is a cause of many reasons. One of them is internal tibial torsion.

Internal tibial torsion is a deformity in the alignment of knee joint causing tibial torsion along its longitudinal axis [1, 2].

**Figure 3:**
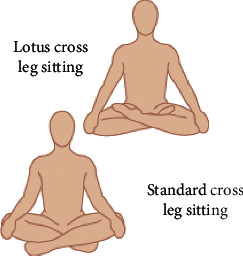
Cross-leg sitting (standard and lotus positions) [2].

The tibia is internally rotated (medial rotation of the shin) in patients with tibial torsion. Diagnosis is made clinically with no need for imaging [3]. The evaluation of tibial rotation depends on several anatomical landmarks, one of them is the tibial tuberosity [2, 4]. In Asians, the upper end of the tibia exhibits more external rotation (i.e., the tibial shaft exhibits more intorsion). So, another reason that may cause lateral adaptation of tibial tubercle is cross-legged posture in Asian culture which is a frequent habit while sitting on the floor, as shown in Figure 3. This posture involves a complex combination of movements of the lower limb joints. At the hip, there is flexion (80° to 101°), external rotation (36° to 52°), and abduction (30° to 57°); at the knee joint, there is flexion (130° to 142°) and internal rotation (17° to 34°), and at the ankle, there is minimal planter flexion [3, 5].

**Figure 4:**
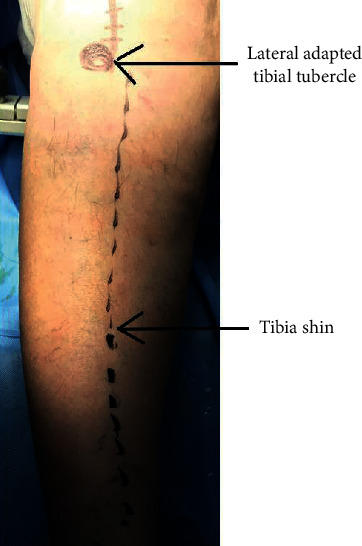
Case with lateral adapted tibial tubercle.

Figure 4 illustrates a case intended for total knee replacement (TKR) with lateral adapted tibial tubercle.

Cinotti in 2013 studied the effect of tibial torsion on total knee replacement and specifically on extramedullary tibial cut guide fixation regarding distal position. They used the center of the intermalleolar distance at the ankle joint as anatomical landmark. Their results state that the center of the ankle joint was shifted laterally by 9–11 mm compared to the proximal axis. Mathematical calculations showed that a 10 mm displacement led to a varus cut of 4.4°. They concluded that the EM guide should be aligned more medially at the distal ankle for cases with tibial torsion to avoid varus implant malalignment [6]. Hernandez-Vaquero and his team in 2018 measured the tibial torsion angle before and after knee replacement to reveal if knee replacement could lessen torsion of the tibia. They found that joint replacement can lessen tibial torsion by approximately 2° (from 17.76° to 15.36° on average) but cannot cancel it [7]. Mizu-uchi et al. in 2006 studied the effect of ankle joint rotation on the tibia cut using the extramedullary cutting guide. They included fifty-three osteoarthritic knees in their study. These joints had external ankle rotation diagnosed using CT scans before the operation. The rotation diagnosis was defined by measuring the angle between proximal tibial plateau axis and distal ankle axis. The extramedullary guide distal end was placed in front of the center of the ankle joint, and the proximal end was placed on the line of the extended anteroposterior axis of the proximal part of the tibia. After the surgery, the tibial coronal alignment was varus (0.5° to 5.1°) for all cases. As a conclusion, if extramedullary alignment guide is used for cases of external ankle rotation, varus alignment of the tibial component can occur [8]. Jörg Lützner et al. in 2010 made an investigation about the tibial tuberosity landmark regarding fixing the cutting guide on the tuberosity medial border or medial third. They found that the mismatch is greater when the cutting guide was fixed according to the medial border than the medial third. They found that the best part of the tuberosity to be followed is the medial third of the tuberosity [1]. During the last few years, researchers investigated solving the tibial alignment problem by using the patient-specific instrumentation (PSI). Kwon et al. in 2017 saw that the surgical operation time was less using the PSI (63.9 ± 13.6 min) compared to CI (82.8 ± 24.9 min), but regarding the alignment, they proved that both techniques conduct to the same results [9]. Kosse et al. in 2018 also proved that both techniques give the same results regarding stability and alignment after they examined 42 patients pre- and postoperatively after 6 weeks and 3 and 12 months and took CT images to evaluate the joint alignment and rotation [10].

In cases of lateral adapted tibial tubercle intended for total knee replacement, an alternative decision should be made regarding coronal tibial alignment for varus fixation to be avoided. One of the three coronal landmarks mentioned in Table 1 would be compromised for this purpose. Tibial tuberosity needs to be followed to ensure correct patellar tracking, since patellar tendon is attached to it. Tibial shin is a parallel guide to the extramedullary rod to ensure perpendicular tibial cut to the tibial shaft. So, midtalar point is the landmark to be neglected by the design presented in this study. This design proposed in this research is a suggested solution for cases of lateral adapted tibial tubercle intended for total knee replacement surgery.

## 2. Subjects

Eight cases of internal tibial torsion were studied with their knees need to be replaced. Their ages were in the range 51–75 years. Six of them were females and two were males. Their MPTAs measured before the knee replacement surgery were on average 87° (80°–94°).

## 3. Method

**Figure 5:**
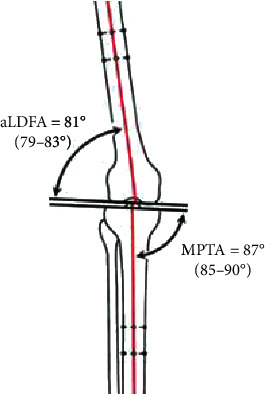
aLDFA and MPTA in normal knee joint.

Independent alignment of the femur and tibia is measured by calculating the angles between their horizontal surfaces with their mechanical axes. These angles are named LDFA (lateral distal femoral angle, normally 87°) and MPTA (medial proximal tibial angle, normally 87°), as in Figure 5.

The aLDFA (81°) corresponds to (anatomical lateral distal femoral angle) which means that it is measured between the anatomical femoral axis and the femoral condylar horizontal surface; adding the 6° to that angle yields the mLDFA (mechanical lateral distal femoral angle). The MPTA (87°) corresponds to (medial proximal tibial angle) meaning that it is measured between the mechanical axis of the tibia and the proximal horizontal tibial plateau [6, 11].

During joint replacement surgery, tibial component generally is implanted perpendicular to the mechanical axis of the tibia in the coronal plane (medial proximal tibial angle (MPTA) 90° rather than 87°) rather than the normal 3° varus avoiding the unintended excessive varus alignment after replacing. The femoral component usually is implanted in 5 to 6 degrees of anatomical valgus and neutral to the mechanical axis (the amount necessary to reestablish a neutral mechanical axis of the limb), so distal femur cut is performed perpendicular to femur mechanical axis and the anatomical lateral distal femoral angle (aLDFA) aimed at 84° rather than 83° for the same reason as tibia. After the surgery, MPTA measurement should be 90° as optimum alignment, ±3° alignment deviation may have to be accepted.

The EM guide is fixed in coronal plane according to three landmarks, tibial tuberosity, tibial shin, and midtalar point. In cases of lateral adapted tibial tubercle, the EM should be placed proximally with lateral tibial tuberosity and parallel to the tibial shin for the reasons that have been cleared in Table 1.

This research included a presentation of eight adapted tibial tubercle cases, a clarification of their MPTAs before and after the surgery, and an illustration of the effect of their tubercle state on the tibial component alignment after the replacement.

Also, the study included designing a new geometrical tool that is meant to be used for those cases intended for knee replacement surgeries. This geometrical tool was proposed as a complementary guide with the extramedullary tibial guide and they both may work together to ensure better tibial cut and improved MPTAs after the replacement.

**Figure 6:**
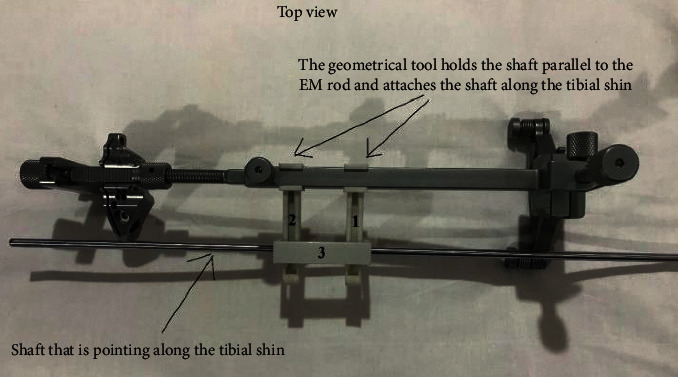
The EM guide with the left cutting block and the geometrical tool prepared to be used for left knees.

**Figure 7:**
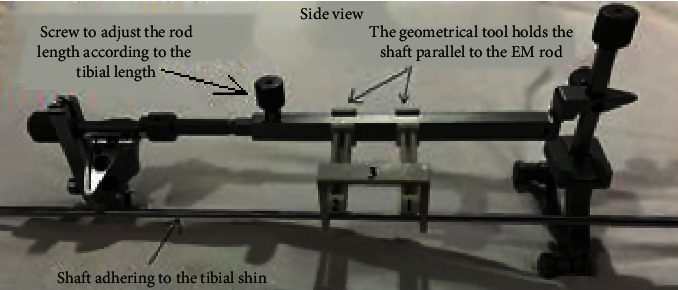
The geometrical tool (numbered 1, 2, and 3) fixed to the original tool.

The working principle of the designed geometrical tool is that it holds another shaft that adheres along the tibial shin and in the same time parallel to the EM guide, as in Figures 6 and 7.

**Figure 8:**
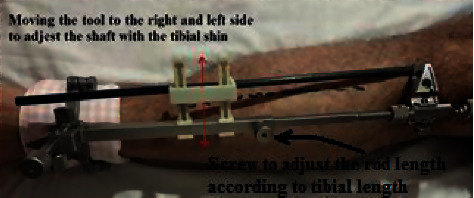
Fixing the tool properly.

First, the surgeon fixes the EM guide to the tibial tuberosity. Then, he fixes the tool to the EM guide and keeps regulating the EM long rod at the ankle junction until the shaft is aligned parallel to the tibial shin, as illustrated in Figure 8.

**Figure 9:**
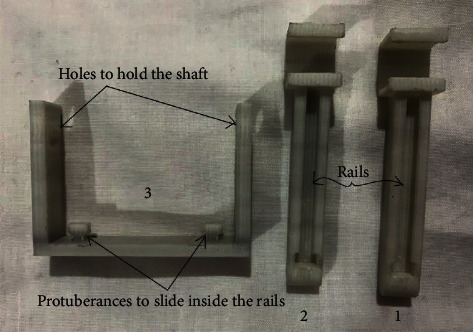
St-PLA prototype of the geometrical tool parts.

The designed tool is composed of three parts. Two of them are holders (numbered 1 and 2 in Figure 9) that hold the shaft bridge. The third part is the shaft bridge (in Figure 9, it is numbered as 3) that holds the shaft along the tibial shin. The tool shown in Figure 9 has been 3D printed as a prototype using the 3D printing material St-PLA.

Each holder has a rail in which the shaft bridge slides along via a protuberance. The purpose of this connection is to accommodate with different stages of lateral tibial tuberosity adaptation.

**Figure 10:**
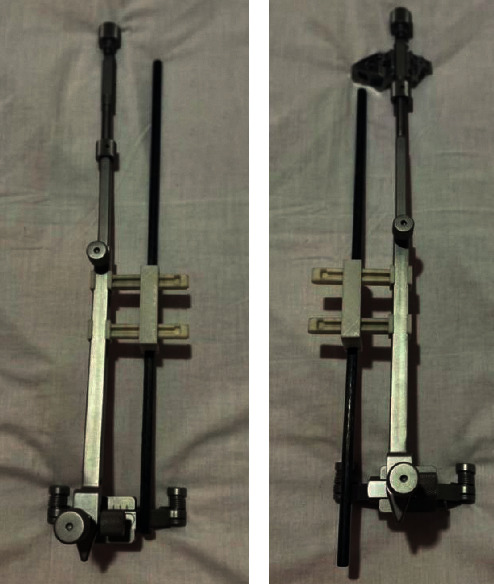
The tool can be fixed for both right and left sides. (a) Right leg. (b) Left leg.

The geometrical tool is designed to be symmetrical for both side usage. It can be used for both the right and left knees, as shown in Figure 10.

## 4. Results

Ten cases intended for primary total knee replacement surgery have been included in this study for tibial tuberosity adaptation clinical examination. Eight out of ten were diagnosed with tibial tuberosity adaptation. The other two cases had normal knees with collinear tuberosity, shin, and midtalar point.

**Figure 11:**
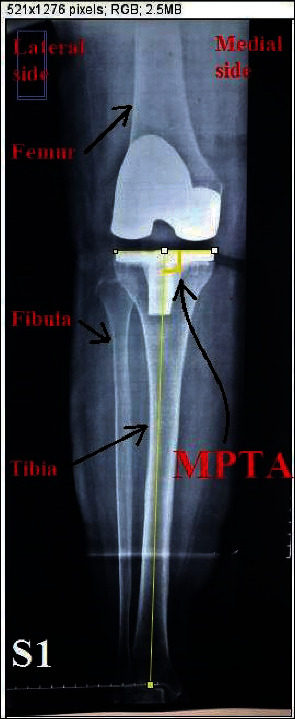
MPTA measurement using ImageJ software.

For all these eight cases, the medial proximal tibial angle (MPTA) has been measured by postoperative X-ray image as an indication for tibial component alignment after the replacement surgery. The X-ray image has been inserted into ImageJ software through which the angle has been measured, as shown in Figure 11.

The angle measurement is done by drawing a line along the tibial plateau and a line along the mechanical axis of the tibia (the line connecting center of the tibial plateau and the midtalar point), and then the angle is the one between these two lines. MPTAs of all eight subjects are shown in Table 2.

## 5. Discussion and Conclusions

Knee replacement reduced the alignment deviation by comparing the MPTA before and after the surgery. Yet, the MPTA measurements after the surgery for the eight studied cases revealed a serious need for alignment improvement in fixing the tibial component of the knee implant. The intended MPTA is 90° for optimum tibial alignment. The mean alignment deviation from the 90° is 2.31° (ranging from minimally 0.54° to maximally 4.5°). Three of the cases (S4, S7, and S8) had an alignment of more than ±3° deviation which is beyond the acceptable range. It can be seen from the results that the gender and knee side have no effect on the MPTA results. Yet, MPTAs of all cases can be improved to reach the optimum 90°. The tool provides a parallel relation between the EM guide and tibial shin, since the holders are designed to be perpendicular to the EM rod and the shaft holder is perpendicular to the shaft. This parallel relation is the needed condition to ensure perpendicular tibial cut to the mechanical tibial axis and, as a result, 90° MPTA. The proposed geometrical tool designed in this study can provide this parallel condition and consequently solve this alignment problem.

## Figures and Tables

**Table 1:** Tibial component alignment in three planes.

Plane of alignment	Anatomical correct alignment	Technical concept to ensure the alignment
Sagittal	Tibial plateau slopes posteriorly 7°–10°	The cutting block has a slope of 7° and the guide rod should be one finger wide from the leg shin proximally and two fingers distally
Coronal	Patella tracked with the tibial tuberosity, leg shin, and midtalar point (MPTA is 90°)	The guide should be aligned to these three landmarks (medial third of tibial tuberosity for best patellar tracking [1], leg shin for a cut that is perpendicular to the tibial mechanical axis, and midtalar point [2])
Transverse	The joint line is the obvious landmark	Shifting the block vertically until the cut on the most defected side (right or left) is 2 mm

**Table 2:** MPTAs of subjects' knees after the surgery.

Subject number	Gender	MPTA after surgery (°)	MPTA before surgery (°)	Knee side
S1	Female	91.871	94	Right
S2	Female	92.286	86	Right
S3	Female	91.443	86	Right
S4	Female	86.927	86	Left
S5	Female	88.755	80	Right
S6	Male	89.567	83	Left
S7	Male	93.415	87	Right
S8	Female	94.562	86	Right

## Data Availability

No data were used to support this study.
